# Exploring MEST: a new universal teaching strategy for school health services to promote positive mental health literacy and mental wellbeing among Norwegian adolescents

**DOI:** 10.1186/s12913-018-3829-8

**Published:** 2018-12-29

**Authors:** Hanne Nissen Bjørnsen, Regine Ringdal, Geir Arild Espnes, Mary-Elizabeth Bradley Eilertsen, Unni Karin Moksnes

**Affiliations:** 1 Department of Public Health and Nursing, Norwegian University of Science and Technology, Postbox 8905, 7491 Trondheim, Norway; 2 NTNU Center for Health Promotion Research, Norwegian University of Science and Technology, Trondheim, Norway

**Keywords:** Mental health literacy, Adolescence, Mental health promotion, School nursing, School health services, Mental wellbeing

## Abstract

**Background:**

Mental health among adolescents is an important public health challenge. School health services perform central public health functions in Norwegian municipalities, where school nurses are uniquely positioned to educate and promote mental health among adolescents. MEST (MEST is not an acronym; MEST is a short version of the Norwegian word for coping) is a newly developed universal working strategy for school health services that aims to promote positive mental health literacy (MHL) and mental wellbeing in the adolescent population. The aim of this study was to investigate the potential outcome mean differences in positive MHL and mental wellbeing between adolescents who participated and those who did not participate in MEST over a school year.

**Methods:**

This study is based on cohort data collected from 357 adolescents (aged 15–21 years) in five Norwegian upper secondary schools at the beginning and end of the 2016/2017 school year. The data were analyzed by describing mean scores and estimating the average treatment effect (ATE) of MEST on positive MHL and mental wellbeing.

**Results:**

Positive MHL increased significantly more among the MEST participants compared to the non-MEST participants (*p* = .02). No significant change in mental wellbeing was found between MEST and non-MEST participants (*p* = .98). Estimating the ATE of MEST on positive MHL, the MEST participants showed a significant 2.1% increase (*p* = .04) in the potential outcome mean of positive MHL compared to the nonparticipants. Estimating the ATE of MEST on mental wellbeing, the girls who attended MEST exhibited a significant 9.7% increase (*p* = .03) in the potential outcome mean of mental wellbeing compared with the girls who did not attend MEST, while no significant change (*p* = .99) was detected among boys or the entire sample of both genders combined (*p* = .12).

**Conclusion:**

This study found a significant ATE of MEST on positive MHL and on mental wellbeing among girls. The results support further investments in studying MEST as a promising work strategy for school health services to promote adolescent mental health. This initial study of MEST may be used as a foundation for investing in future evaluations of MEST.

## Background

In recent years, mental health among adolescents has received considerable attention as a public health concern that is important to address both internationally and in Norway [1–4]. Since the 1986 Ottawa Charter for Health Promotion [5], the focus of public health has shifted from disease prevention only to including health promotion. Scholars advocate for the importance of appropriate attention to mental health within the field of health promotion [6]. Like health promotion, mental health promotion involves the process of enabling people to increase control over, and improve their mental health; supporting people in adopting and maintaining healthy lifestyles. It seeks to foster and support individual and social resources, competencies and psychological strengths to benefit mental health and wellbeing, complementary to a focus on preventing mental disorders [7]. What mental wellbeing is or involves is often considered a highly individual matter; it can be argued to be an individual preference. However, there are known commonalities that are important for mental wellbeing. In the current study, Clarke et al.’s definition is the basis for the understanding of mental wellbeing: “a positive and sustainable mental state that allows individuals to thrive and flourish” ([2, 8], p.).

Adolescents constitute an important population from a public health perspective. Adolescents are expected to acquire knowledge and abilities that will be important for their eventual development into a healthy adult population that can assume adult roles in society. Adolescence is considered a vital transitional period in life that is associated with challenges as well as opportunities for growth, development and health promotion [9]. Furthermore, adolescence is a critical phase for building a foundation for a future healthy population [10, 11]. Because approximately 20% of adolescents report that mental health problems affect their daily life [1, 2], adolescence is an important period in the life course for public health strategies addressing mental health. Public health work strategies and programs that promote good mental health also help to prevent mental illness [12]. Hence, the promotion of good mental health and the prevention of mental illness are considered complementary strategies.

Health literacy is emphasized as an important social determinant for equity in health and is considered necessary for participation in health promotion activities. Broadly speaking, health literacy involves the ability to make sound health decisions [13] and is often studied as an outcome of health education [14]. Mental health literacy (MHL) originates in health literacy and is an emergent area of research in the field of health promotion. It has been identified as an important determinant of both individual and public mental health [15–19]. MHL refers to an individual’s knowledge and ability required to make sound mental health decisions in everyday life [16]. MHL is a relatively new concept in health promotion research, and multiple definitions and models have been identified [20]. Recently, MHL has been defined by Kutcher et al. as consisting of the following four components:

“(1) Understanding how to obtain and maintain good mental health; (2) understanding mental disorders and their treatments; (3) decreasing stigma related to mental disorders; and (4) enhancing help-seeking efficacy (knowing when, where and how to obtain good mental health care and developing competencies needed for self-care)” [17].

The first component of MHL in Kutcher et al.’s definition is referred to in this study as positive MHL (1), understanding how to obtain and maintain good mental health. This component (1) is essential from a health promotion perspective in which the focus is on knowledge of good mental health rather than on mental disorders. Previous research investigating MHL has mainly focused on the three latter components in Kutcher et al.’s definition: the recognition of mental disorders; help-seeking efficacy and help-seeking strategies (e.g., [16, 18, 21–24]). Among adolescents, positive associations have been found between low MHL and mental illness, particularly anxiety and depression [25]. To the best of the authors’ knowledge, only one study has investigated the relationship between MHL and mental wellbeing. In that study, positive MHL demonstrated a significant and positive relationship with mental wellbeing [26].

Adolescents spend a large amount of their time at school, and universal mental health promotion in the school setting using a whole school approach is recognized as particularly effective for mental health promotion in this population [27, 28]. School health services represent an essential part of the whole school approach and play an important role in the field of public health by providing easy access and universal healthcare services to the adolescent population [29, 30]. School nurses within school health services are uniquely positioned and are expected to promote good mental health at the population level, provide mental health education, and address diverse health problems in the adolescent population [29, 30].

Several school-based programs aimed at mental health promotion are available internationally [28] and in Norway [31]. On behalf of the Norwegian Directory of Health, the Regional Centre for Child and Youth Mental Health and Child Welfare (RKBU North) identified and described six interventions available for Norwegian schools that target mental health promotion and have “sufficient documentation of an effect” (“Respekt”, “VIP”, “Venn1”, “Alfa”, “Zippys venner”, “Olweus”, and “PALS”) [31]. None of these identified programs have been found to address the role of school health services or school nurses in mental health promotion. Furthermore, the intervention “Mental health for everyone” is designed to promote MHL among Norwegian adolescents; however, it is only labeled “probably effective” according to RKBU’s systematic reviews [31]. In one study, “Mental health for everyone” had a positive impact on adolescents’ MHL by increasing recognition of mental disorders, prejudice and knowledge regarding where to seek help [21]. Positive MHL was not included in the study and has not been identified in any studies addressing MHL. There is an explicit need for research that investigates the effectiveness of school-based MHL programs [18].

### MEST – a school-based MHL working strategy for school health services

Consistent with national regulations and professional guidelines [29, 32], and with financial support from the Norwegian Directory of Health, school health services in Trondheim, Norway, have developed and implemented a universal health education working strategy in upper secondary schools named MEST. MEST has a salutogenic foundation. It was developed in 2014 and has not been previously described or evaluated. The core aim of MEST is to increase adolescents’ positive MHL and to provide resources for mental wellbeing by focusing on adolescents’ assets and the promotion of personal and contextual factors for *good* mental health. MEST offers open school seminars, classroom seminars and smaller group discussions with adolescents and is based on voluntary student participation. Thus not all students at a school offering MEST will have participated in MEST. School health services deliver targeted seminars and discussion groups throughout the school year based on the results of an anonymous digital survey that is completed by students at the beginning of each school year. The seminar topics may include, but are not limited to, normal emotional variations, sleep hygiene, stress management, relaxation techniques, body image, self-esteem, and aspects related to autonomy (e.g., making decisions based on one’s own will and recognizing personal limits).

Although the seminars provided at each school may differ, these seminars are based on a common framework that includes the following: 1) a theoretical understanding of the seminar topic, 2) practical age-appropriate examples, and 3) providing adolescents with at least one specific and useful tool related to the subject of the seminar (Holmen N. Description of MEST: a work strategy for school nurses in mental health promotion among adolescents. 2016. Personal written and oral communication, recipient: HN Bjørnsen, 2016 document). MEST differs from previous interventions that aim to promote mental health in schools, such as “Mental health for everyone” [21], because MEST is a systematic work strategy that focuses on promoting *good* mental health and coping with normative stressors and emotional variations instead of preventing mental disorders.

Given the increase in mental health problems in the adolescent population and the importance of mental health promotion initiatives at school, the identification, implementation and evaluation of effective mental health interventions are essential [33]. The recognition of school health services as an important component in a whole school approach highlights the importance of evaluating mental health-promoting actions initiated by school health services. An initial assessment of MEST is important to determine whether further investment in more rigorous studies of this work strategy is worthwhile. Moreover, further appraisal of whether MEST has the potential for continued evolvement and implementation as a preferred way of systematizing school health services’ mental health-promoting work is important, both for advancing evidence-based practices and for documenting the outcomes of new mental health promotion initiatives.

### Aim

The aim of this study was to investigate the potential outcome mean (POM) differences in positive MHL and mental wellbeing between adolescents who participated in MEST and adolescents who did not participate in MEST.

## Methods

### Participants and procedure

The study participants included a cohort of 357 adolescents aged 15–21 years in mid-Norway. The adolescents were sampled from five upper secondary schools where school health services used the MEST working strategy during the 2016/2017 school year. The schools also offer various activities for health promotion throughout the school year, such as a public health day, an adolescent health day and a “VIP” program (see introduction) as part of the regular operations of Norwegian upper secondary schools. The schools offer a broad variety of vocational and general courses and represent typical Norwegian upper secondary schools. The size of the schools varies from 260 to 1087 students, and the gender distribution is even. Most of the adolescent participants were born in Norway and self-reported that they had parents with higher education and perceived their family had a good financial situation (Table 1). Table 1 further describes the study population by MEST participation.

**Table 1:** Descriptive statistics of the baseline (T1) cohort and distribution of the MEST and non-MEST participants

	Entire Cohort		MEST Participants		Non-MEST Participants	
	N	Percent (%)	N	Percent (%)	N	Percent (%)
	357		109		248	
Gender^a^						
Female	188	53	78	72	110	44
Male	166	46	30	27	136	55
Missing	3	< 0.1	1	< 0.1	2	< 0.1
Age (years)						
15	2	< 0.1	–	–	2	< 0.1
16	151	42	44	40	107	43
17	104	29	36	33	68	27
18	72	20	27	25	45	18
19	23	6	1	1	22	9
20	1	< 0.1	1	1	1	< 0.1
21	1	< 0.1	–	–	1	< 0.1
Missing	3	< 0.1	–	–	2	< 0.1
Education						
General studies	226	63	63	58	163	66
Vocational studies	128	36	45	41	83	33
Missing	3	< 0.1	1	1	2	< 0.1
Parental education^b^						
Primary or lower secondary school	19	5	4	4	15	6
Upper secondary school	81	23	26	24	55	22
University up to 4 years	116	32	32	29	84	34
University more than 4 years	83	23	33	30	50	20
Unknown	47	13	12	11	35	14
Missing	11	3	2	2	9	4
Family finances^c^						
Good	268	75	82	75	181	73
Neither good nor bad	64	18	22	20	51	21
Bad	19	5	2	2	13	5
Missing	6	2	3	3	3	1
Parents live together						
Yes	230	64	67	61	162	65
No	124	35	41	38	84	34
Missing	3	1	1	1	2	< 0.1
Born in Norway						
Yes	336	94	104	95	232	94
No	16	4	3	3	13	5
Missing	5	1	2	2	3	1

^a^Significant difference between MEST and non-MEST participants

^b^ Student report of parents’ highest education. Assessed by asking about each parent; the mean score of the mother and father is presented

^c^ Student perception of family finances

The principals of the designated schools provided informed consent for data collection. The schools were asked to participate in the study because the school nurses at these schools used the MEST working strategy for mental health promotion throughout the 2016/2017 school year. A study-specific questionnaire was administered by the teachers at the beginning and end of the 2016/2017 school year (T1: September 2017 and T2: April–June 2017). MEST was offered at the schools between the data collection time points (T1 and T2). Five schools originally agreed to participate in the study, but one school withdrew before the T2 assessment. At baseline T1, by the teachers’ decisions, the questionnaire was administered to 2145 of the 3281 (65.4%) students at the five schools, and 2087 students responded with usable information (T1 response rate was 97.3%). At T2, again by the teachers’ decisions, the questionnaire was administered to 1127 of the 2811 (40.1%) students at the four schools, and 1054 students responded with usable information (T2 response rate was 93.5%). The teachers were encouraged to administer the questionnaire by their principal; however, each teacher decided whether to administer the questionnaire. Students of teachers who decided not to administer the questionnaire were thus not given the opportunity to participate in the study. To match the adolescents from baseline T1 to T2, three questions were asked in which the first two letters of each answer created a six-letter code used to anonymously follow the student cohort. The six-letter code allowed for 34.2% (361) of the students to be matched from T1 to T2. The main reason for the low matching rate was that teachers might not have administered the questionnaire to the same students at T1 and T2, resulting in some students only having the opportunity to answer at baseline T1 or at T2. Of the 361 students who were matched, 357 (33.8%) students were aged 15–21 years and constituted the net sample. Of these 357 students, 248 (69%) students reported that they did not attend or did not know whether they had attended MEST over the last school year, whereas 109 (31%) students reported that they had attended MEST. Of the MEST participants, 79 (72%) students were females and 30 (27%) students were males (Table 1).

Informed consent forms were used for participants aged ≤15 years (parental consent is required by law), whereas participants aged > 15 years consented by completing the questionnaire [34]. Regardless of age, all students received the same information. An informational video was available on the schools’ e-learning platforms (e.g., “it’s learning”) to inform students about participation in the study and to emphasize that participation was voluntary and anonymous. The same information was provided on the survey’s first page and read aloud by the teachers prior to survey administration. This study was approved by the Regional Committee for Medical and Health Research Ethics (REK midt 2014/1996).

### Measures

The two outcome variables (i.e., positive MHL and mental wellbeing) were predetermined because MEST explicitly aims to promote positive MHL and mental wellbeing.

**Positive MHL** was measured by the 10-item Mental Health Promoting Knowledge (MHPK-10) scale [35]. The MHPK-10 scale is a one-dimensional instrument consisting of statements related to factors important for good mental health [35]. The respondents are asked to rate each item on a six-point scale ranging from 0 (“don’t know”) and 1 (“completely wrong”) to 5 (“completely correct”). Higher mean scores indicate a higher level of knowledge (range 0–5). The MHPK-10 scale was recently determined to be a valid and reliable measure of positive MHL among Norwegian adolescents [35] and had a Cronbach’s *α* of .81 at T1 and .83 at T2.

#### Covariates of positive MHL

The parents’ education level and student grade level were included as covariates in the analysis of MEST’s ATE on positive MHL. Positive MHL may be influenced by the parental education level because positive MHL is considered an outcome of mental health education, and parental education level is a well-known predictor of children’s educational outcomes [36]. Furthermore, the students’ grade level was assumed to potentially influence positive MHL because the grade level may indicate the general level of knowledge; the longer adolescents have studied, the more knowledge they are expected to possess.

*Parental education level* was assessed by one item, i.e., “What is your parents’ highest level of education?” The response options included (1) primary or lower secondary school, (2) upper secondary school, (3) university for up to 4 years, and (4) university for more than 4 years.

**Mental wellbeing** was assessed by the Short Warwick-Edinburgh Mental Wellbeing Scale (SWEMWBS). The SWEMWBS is a short version of the Warwick-Edinburgh Mental Well-being Scale (WEMWBS) that measures subjective wellbeing and psychological functioning using seven items scored on a five-point Likert scale ranging from 1 (“not at all”) to 5 (“all the time”). Higher mean scores indicate greater wellbeing (range 1–5) [37]. The SWEMWBS allows for the monitoring of the mental wellbeing of the general population and is validated for use among young people [38]. In this study, the Cronbach’s *α* of the SWEMWBS was .88 in the T1 sample and .91 in the T2 sample.

#### Covariates of mental wellbeing

Mental wellbeing can be considered a highly individual matter that may be influenced by a number of factors. However, some variables are known to commonly affect mental wellbeing. The following variables were adjusted for in the model assessing mental wellbeing: gender [39], symptoms of anxiety and depression [40], self-rated health [41], loneliness [42], school-related stress [43], health literacy (only assessed at T2) [44, 45], and social inequalities, which were best represented by the variable of family finances [46].

*Anxiety and depression* were assessed using the 10-item Hopkins Symptom Checklist (HSCL-10) [47, 48]. Six of the 10 items on the scale are related to depression, whereas four items are indicators of anxiety [49]. The response scale ranges from 1 (not at all) to 4 (extremely), and higher mean scores indicate higher severity of anxiety and depression symptoms. The Cronbach’s *α* of the HSLC-10 was .92 in the T1 sample and .93 in the T2 sample. The HSCL-10 is a validated and frequently used scale that measures anxiety and depression symptoms among adolescents [48].

*Self-rated health* was assessed using the following item: “How is your current health?” The students responded on the following scale: (1) very poor, (2) poor, (3) neither poor or good, (4) good, and (5) excellent. This item has been previously found to be satisfactory for use among adolescents [41].

*Loneliness* was assessed by one item covering the frequency of feeling lonely, i.e., “Do you ever feel lonely?” The response options included (1) never or almost never, (2) rarely, (3) sometimes, (4) regularly, and (5) almost all the time.

*Stress* was assessed using the school-related stress dimensions of the Norwegian version of the Adolescent Stress Questionnaire (ASQ-N) [50, 51]. Each of the four dimensions in the ASQ-N assessing school-related stress included four items. The 16-item scale is rated on a five-point Likert-type scale ranging from 1 (not at all stressful or irrelevant to me) to 5 (very stressful). Higher mean scores indicate higher stress levels (range 0–5). The internal consistency and construct validity of the ASQ-N have been tested among adolescents [50, 51]. The Cronbach’s α in the present study was .93 at T1 and .92 at T2.

*Health literacy (HL)* was measured with the Health Literacy for School-Aged Children (HLSAC) scale. The HLSAC is based on the conceptualization of health literacy proposed by Paakkari and Paakkari [52]. The HLSAC scale consists of 10 items, and respondents are asked to rate the degree to which each item represents their opinion on a scale ranging from 1 (“not at all true”) to 4 (“absolutely true”) [53]. The HLSAC scale has been shown to be a valid measure of adolescent health literacy in a Nordic country [52]. The Cronbach’s *α* of the HLSAC was .88 at T2. The HLSAC was added before the second data collection.

The *background variables* used in this study included gender, student grade level, field of study (grouped into general and vocational studies), parents’ living status (grouped into living together or not), whether the respondent was born in Norway, years living in Norway, parental education and family finances*.*

*Family finances* were measured by the following item: “How has your family’s financial situation been during the past two years?” The students responded on the following scale: (1) we have had a poor financial situation the whole time, (2) we have more or less been in a poor financial situation, (3) we have been in neither a poor nor a good financial situation, (4) we have more or less been in a good financial situation, and (5) we have been in a good financial situation the whole time.

*Participation in MEST* was measured by a single question (“Did you participate in MEST seminars, lectures or groups over the last school year?”). The response options included (1) “no”, (2) “yes” and (3) “don’t know”. Participants who responded (3) “don’t know” were coded as not participating and assigned the value 1 for “no”.

### Statistical methods

STATA version 14.2 (StataCorp. 2015, Stata Statistical Software: Release 14, College Station, TX: StataCorp LP [54]) was used to perform the descriptive statistics and statistical analyses. T-tests and Chi-square tests of independence were performed to evaluate the baseline differences between the MEST and non-MEST participants in terms of the background variables (Table 1). Independent t-tests with equal variances were conducted to assess the mean group differences (MEST participants vs. non-MEST participants) in age, family finances and years living in Norway. Cohen’s *d* was used to interpret the effect sizes [55]. Chi-square tests of independence were performed to assess the group differences (MEST participants vs. non-MEST participants) in gender, line of study, parental education level and parents’ marital status. Furthermore, in addition to the included covariate scales of anxiety and depression, self-rated health and HL, the mean scores and confidence intervals (CI) of the outcome measures of positive MHL and mental wellbeing were examined. Paired samples t-tests with equal variances were conducted to assess the mean group differences between the baseline and T2 scores of the outcome variables positive MHL and mental wellbeing. The data were stratified by gender to examine potential gender differences.

#### Treatment effect modeling

Linear treatment effect modeling with augmented inverse probability weighting (AIPW) and double robust estimators were used to estimate the average treatment effect (ATE) of MEST based on MEST participation (i.e., treatment). Treatment effect modeling is used to describe the observed statistical relationship using observational data based on potential treated and untreated responses [56, 57]. AIPW models both the treatment and outcome and maintains consistency even if one of the models is mis-specified [58]. Doubly robust estimators are recommended as the preferred estimators for estimating the ATE in non-normally distributed data [59] and are used because of the potential ceiling effects observed in the outcome variables positive MHL and mental wellbeing. The conditional independence of MEST on the outcomes is assumed after adjusting for potential confounders. The percentage effects were calculated to display the ATEs relative to the baseline POMs (non-MEST participants). In all analyses, we adjusted for the baseline measure (T1) of the analyzed outcome and the predetermined covariates. The data were analyzed for both genders separately and combined to explore potential gender differences in the impact of MEST. The significance level was set at *p* ≤ .05.

#### Missing data

The data were assessed for patterns of missing values. For the main variable positive MHL (measured by the MHPK-10), 1.65% (item 1) to 3.1% (item 4 and 10) of the values were missing. Little’s [60] missing completely at random (MCAR) test was used to test the hypothesis that the values were missing completely at random (*p* ≥ .05) [60]. Little’s MCAR test supported the hypothesis that the values were missing completely at random for MHPK-10 (*p* = .36) and SWEMWBS (*p* = .70) [38]. Cases were deleted listwise.

## Results

### Baseline differences between MEST and non-MEST participants

The results of the Chi-square test of independence showed a significant interaction (χ^2^ (1) = 20.77, *p* = *≤* .01) between gender and MEST participation; significantly more girls than boys chose to participate in MEST. No significant interaction (χ^2^ (1) = 2.15, *p* = .14) was observed between the line of study and MEST participation. Furthermore, no other significant differences were found between the group that participated in MEST and the group that did not participate with respect to family finances, years lived in Norway and age (Table 2).

**Table 2:** Mean group differences in background variables between the MEST and non-MEST participants

		MEST Participants	Non-MEST Participants			
	N	M (SD)	M (SD)	t-test	*p*-value	Cohen’s *d*
Family finances^a^	351	3.69 (0.07)	3.62 (0.33)	−0.75	0.77	0.29
Years lived in Norway	321	17.0 (0.23)	17.3 (0.12)	0.66	0.25	1.6
Age	357	17.6 (0.76)	17.6 (0.47)	−0.02	0.51	0

^a^ Student perception of family finances. Poor = 1, Good = 5

### Descriptives and differences in the mean scores of the main variables from baseline (T1) and T2

Mean scores and CIs of the primary measures of positive MHL and mental wellbeing and the covariate scales of anxiety and depression, self-rated health and HL are presented by MEST participation in Table 3. The results showed an overall increase in positive MHL among both MEST and non-MEST participants between assessment points and that the MEST participants had a significantly larger increase in positive MHL than the non-MEST participants (M = 4.56, SD = 0.04) to T2 (M = 4.65, SD = 0.03) scores (t (105) = − 2.15, *p* = 0.02). Girls’ baseline scores on positive MHL was higher than boys’ baseline scores on positive MHL. The positive MHL of the boys who participated in MEST increased, whereas the positive MHL of the boys who did not participate in MEST slightly decreased. For the girls, positive MHL increased among MEST participants and was stable among non-MEST participating girls (Table 3). In mental wellbeing, no significant change in mental wellbeing was found between MEST and non-MEST participants; a non-significant decrease in scores were observed over the school year from T1 (M = 3.53, SD = 0.07) to T2 (M = 3.40, SD = 0.08) conditions (t (100) = 2.04, *p* = 0.98); and the scores decreased less among the girls who participated in MEST than among the girls who did not participate (t (172) = − 1.2, *p* = 0.12). In the boy cohort, mental wellbeing decreased less if the students did not participate in MEST. However, the difference in boys’ mental wellbeing means between baseline T1 and T2 was not statistically significant when comparing boys’ mental wellbeing scores at T1 (M = 3.8, SD = 0.06) to boys’ mental wellbeing at T2 (M = 3.69, SD = 0.07) (t (230) = 13.1, *p* = 0.95). The boys reported higher baseline mean scores of mental wellbeing than the girls did (Table 3). The anxiety and depression scores increased over the school year and increased slightly more among the MEST participants than among the non-MEST participants. The self-rated health scores decreased between assessment points, with similar scores observed in both MEST and non-MEST participants (Table 3). The mean score differences were statistically tested for the main variables of positive MHL and mental wellbeing.

**Table 3:** Mean scores (CI) of included scales at baseline T1 and T2. Outcome variables are distributed by gender and MEST participation

	Baseline T1				T2			
	MEST participants		Non-MEST participants		MEST participants		Non-MEST participants	
Outcome	N	Mean score (CI)	N	Mean score (CI)	N	Mean score (CI)	N	Mean score (CI)
Positive MHL^a^	106	4.56 (4.48–4.64)	245	4.47 (4.43–4.55)	107	4.65 (4.58–4.71)	222	4.53 (4.45–4-60)
Positive MHL (girls)	76	4.57 (4.47–4.67)	106	4.64 (4.57–4.71)	79	4.65 (4.58–4.73)	102	4.63 (4.56–4.71)
Positive MHL (boys)	29	4.52 (4.36–4.68)	126	4.45 (4.33–4.56)	30	4.62 (4.49–4.76)	119	4.43 (4.31–4.55)
Mental wellbeing^b^	106	3.53 (3.39–3.66)	229	3.59 (3.49–3.69)	104	3.40 (3.24–3.56)	219	3.47 (3.36–3.57)
Mental wellbeing (girls)	78	3.43 (3.28–3.58)	103	3.35 (3.15–3.56)	74	3.33 (3.16–3.51)	100	3.20 (3.07–3.33)
Mental wellbeing (boys)	27	3.84 (3.55–4.13)	124	3.80 (3.67–3.92)	30	3.55 (3.20–3.91)	118	3.69 (3.54–3.83)
Covariates								
Anxiety and depression^c^	102	1.82 (1.68–1.95)	228	1.70 (1.60–1.79)	198	1.97 (1.82–2.13)	219	1.75 (1.65–1.84)
Self-rated health^d^	108	3.95 (3.79–4.12)	244	4.05 (3.95–4.16)	109	3.83 (3.66–3.99)	225	3.82 (3.70–3.95)
HL^e^	–	Not measured T1	–	Not measured T1	101	3.25 (3.16–3.34)	210	3.11 (3.25–3.37)

Cases were deleted listwise

^a^Positive MHL was measured by the MHPK-10

^b^Mental wellbeing was measured by the SWEMWBS

^c^Anxiety and depression were measured by the HCSL-10

^d^Self-rated health was measured by a single item

^e^HL was measured by the HLSAC

### Average treatment effect (ATE) of MEST

A significant ATE of MEST was found on positive MHL (Table 4) when both genders were combined, indicating that on average and after adjusting for the baseline T1 scores of positive MHL and including potential confounders (parental education level, grade level and years living in Norway), all participants in this sample who participated in MEST scored 2.1% higher on positive MHL than all adolescents in the sample who did not participate in MEST. This finding represents a statistically significant increase in positive MHL in both genders. However, after stratifying by gender, no significant ATE of MEST was found in either gender separately (results not shown). A non-significant ATE of MEST on mental wellbeing was found. However, after stratifying by gender, a significant ATE of MEST on mental wellbeing was found in the girls, and a non-significant and negative ATE of MEST on mental wellbeing was found in the boys (Table 4). The percentage change reflects the change in the ATE between the participating and nonparticipating adolescents’ POM, indicating that if none of the adolescents in the current sample had participated in MEST, they would have had a mean score of 4.54 on positive MHL (POM of those who did not participate). If all adolescents in the sample had participated in MEST, they would have had a mean score of 4.63 on positive MHL (POM of those who participated), resulting in an ATE of .10 and a percentage increase of 2.1%. Both the ATE and percentage increase were statistically significant. The mental wellbeing POM of the participating girls was 3.48, while that of the nonparticipating girls was 3.17, resulting in a statistically significant ATE of 0.31 and a percentage increase of 9.7%.

**Table 4:** Estimates of the ATEs of MEST on positive MHL and mental wellbeing. Mental wellbeing is stratified by gender

Outcome	POM^¥^ of participants (N)	POM^¥^ of nonparticipants (N)	ATE^+^	ATE^+^ 95% CI	*p* of ATE^+^	% change	% change CI	*p* of %
Positive MHL^a,1^	4.63 (99)	4.54 (205)	0.10	0.01–0.20	0.04*	2.1	0.2-4.4	0.03*
Mental wellbeing^b,2^	3.60 (78)	3.40 (167)	0.20	−0.05-0.46	0.120	6.0	−1.6-13.5	0.123
Mental wellbeing (girls)	3.48 (63)	3.17 (81)	0.31	0.03–0.58	0.028*	9.7	0.8-18.5	0.031*
Mental wellbeing (boys)	3.70 (15)	3.71 (101)	−0.005	−0.61-0.6	0.988	0.01	−16.5-16.2	0.988

*Significant at *p* ≤ 0.050

Total *N* = 340; participated *n* = 109; did not participate *n* = 229. Cases deleted listwise

^a^Positive MHL = Positive mental health literacy measured by the MHPK-10

^b^Mental wellbeing was measured by the SWEMWBS

^+^ATE = average treatment effect

^¥^POM = potential outcome mean

^1^Values were adjusted for baseline positive MHL, parental education level, grade level and years living in Norway

^2^Values were adjusted for baseline mental wellbeing, anxiety and depression, gender, physical health, HL, loneliness, family finances and school-related stress

## Discussion

This study compared positive MHL and mental wellbeing between two groups of adolescents, MEST and non-MEST participants. The average treatment effect (ATE) was applied to start the process of understanding MEST and to guide future potential investment in more rigorous and resource-intensive evaluations of MEST. This initial evaluation of MEST shows a tendency toward a small but significant increase in positive MHL among MEST participants compared to non-MEST participants. The results showed an increase in positive MHL and a decrease in mental wellbeing in both MEST and non-MEST participants between assessment points. However, when modeling the treatment effect of MEST on mental wellbeing, there was an estimated and significant positive treatment effect of MEST on girls’ mental wellbeing and a significant positive treatment effect of MEST on positive MHL for both genders combined.

### Methodological considerations

The assessment of mental health promotion initiatives and interventions is challenging because attributing outcomes to the initiatives is difficult and because choosing evaluation methods and outcome measures always involves balancing conceptual, ethical and clinical considerations [61]. The non-standardized nature of the work strategy is a strength of MEST, making it adaptable to the current needs of a particular population based on annual local surveys. However, due to this non-standardization, MEST is challenging to evaluate. The current study applied treatment effect modeling to compare the observed statistical relationship and scores of positive MHL and mental wellbeing between MEST participants and non-MEST participants. The analyses were intended to investigate whether pursuing more rigorous evaluations of MEST is reasonable and to establish a foundation for future evaluations, such as a randomized controlled trial.

Over a school year, one would expect students to mature and possibly increase their knowledge base in general. Therefore, one may expect that positive MHL might increase for both groups as the adolescents grow older, mature and learn over a school year. This may serve as one explanation for both groups’ increase in positive MHL, in which the MEST group’s scores on positive MHL increased more than the non-MEST group’s scores (Table 3). Because MEST aims to increase adolescents’ positive MHL, it is sensible that the MEST group’s positive MHL increases more than that of the non-MEST group if MEST succeeds at impacting adolescent positive MHL. Mental wellbeing decreased over a school year for both groups, and among boys, mental wellbeing decreased less if the students did not participate in MEST. This finding raises the question of whether MEST has a negative impact on boys’ mental wellbeing. The difference was not statistically significant. Thus, this decrease may be related to normal variations and explained by a number of factors that the current study is not able to detect. The descriptive statistics showed that there was an overall decrease in both groups in mental wellbeing and self-rated health and an increase in anxiety and depression symptoms over a school year (Table 3). One explanation in relation to the descriptive statistics results is that baseline T1 data were collected during the fall semester after the adolescents returned from their summer break, while the T2 data were collected during the spring when adolescents were approaching their final exams. This might potentially affect the adolescents’ reporting on variables such as mental wellbeing, self-rated health and anxiety and depression symptoms. However, this does not explain why anxiety and depression symptoms were reported to be higher among MEST participants than among non-MEST participants.

### Gender differences

A potential explanation for finding higher mean scores on anxiety and depression among MEST participants than among non-MEST participants might be related to gender differences: There is found a predominance of depression among girls compared to boys in adolescence [62], and there are more girls (72%) in the MEST group than in the non-MEST group (44% girls). For some time, evidence has highlighted a greater predominance also of the development of depression in girls than in boys in adolescence [62]. Research has also demonstrated that girls’ mental wellbeing scores are lower than boys’ scores but appear to increase during adolescence, whereas boys are found to have higher and more stable scores on mental wellbeing measures throughout adolescence [39], suggesting that girls’ mental wellbeing might be more prone to internal and external developmental factors throughout adolescence. This finding may serve as a possible explanation for the ability of MEST to influence girls’ mental wellbeing more than boys’ mental wellbeing. The significant improvement in the ATE of MEST on mental wellbeing found among the girls in the current sample was not reflected by a significant difference in the ATE by gender on positive MHL, indicating that factors other than positive MHL could explain the differences in mental wellbeing between boys and girls. Previous studies investigating gender differences in MHL focusing on recognizing disorders have demonstrated that gender differences in MHL are not as prominent as previously thought [25]. The results of the present study are consistent with these previous findings. The gender differences in the ATE of MEST on mental wellbeing was not reflected in the levels of positive MHL. Another explanation for the gender differences in this sample might be that MEST is more appealing to girls. This explanation is evident considering the attendance rates based on gender; in this sample, significantly more girls than boys chose to attend MEST. However, considering the plain mean scores, compared with the boys who did not attend MEST, the boys who attended MEST were found to have an increase in positive MHL (Table 3). This finding indicates that the boys who attended MEST developed higher positive MHL throughout the school year than boys who did not attend MEST. However, these changes cannot be attributed to MEST based on the plain mean scores, and further research is needed to understand these differences.

### Statistical and clinical significance

The 2.1% increase found in positive MHL among adolescents who attended MEST compared to those who did not attend MEST is statistically significant but small. From a public health perspective, a small increase might have a large public health impact and, thus, may have clinical significance. If we successfully move populations in a positive direction, there might be a greater overall public health impact than the impact that can be detected at the individual level. In addition, there is a possible ceiling effect in the MHPK-10 measure of positive MHL [35], indicating the possibility that we cannot distinguish among higher levels of positive MHL using this measure and resulting in the potential underestimation of the impact of MEST on positive MHL. Thus, the impact may be even larger than the impact that this study can detect. Furthermore, a 6.0% statistically non-significant ATE of MEST on mental wellbeing for both genders combined and a 9.7% statistically significant ATE of MEST on girls’ mental wellbeing may be clinically interesting for practitioners and researchers who aim to promote mental wellbeing in the adolescent population.

### Implications

Schools are considered crucial settings for promoting mental health, and school health services (i.e., health services specializing in health promotion for individuals aged 0–20 years located at schools) are well positioned to promote good mental health in the adolescent population. This study adds to the evidence regarding the use of universal mental health-promoting strategies, such as MEST, focusing on assets for positive mental health in the adolescent population. Investing in adolescents’ mental health using evaluated and documented approaches to promote mental health may yield short- and long-term benefits on positive development for adolescents that may extend throughout the life course [63]. This study may serve as a foundation for the further process of evaluating a reorientation of school healthcare services to include a focus on universal mental health promotion in schools by concentrating on positive MHL and mental wellbeing. Using universal strategies, such as MEST, that focus on positive mental health as a part of the whole school approach is consistent with evidence that clearly indicates that a positive school ethos in which school health services are naturally embedded is associated with students’ health [64]. Universal strategies are also supported by adolescents’ overall expression of the need for more knowledge about mental health [65].

Traditionally, school health services and school nurses in Norway have provided individual-focused care to adolescents in upper secondary schools. This practice may serve as a barrier to implementing universal strategies in a school health setting. Individual-focused care is familiar and easy to manage, probably because system barriers are not as apparent during student encounters [66] as they might be when integrating universal mental health-promoting initiatives in schools that target the entire student population, such as MEST. Moreover, there is an embedded resource and priority question; individual care traditionally is (and, to some extent, should be) a priority among school nurses. This study adds to the discussion of the responsibilities of school health services and school nurses with respect to public health and health promotion in schools. However, based on the methods used, the results of this study should not be used to recommend MEST but should rather be used to recommend further investments in more rigorous and resource-intensive evaluations of MEST. Thus, this study can be considered an important foundation for further evaluations of MEST.

### Strengths and limitations

The major strengths of the current study are the use of longitudinal data and the high response rate for both baseline T1 and T2. The longitudinal data enabled the adjustment of the baseline values of the outcome variables for each individual participant. Validated instruments and recognized single items were used, although positive MHL was measured by a newly developed measure that has been reported to have potential ceiling effects [26]. Nevertheless, the MHPK-10 has been shown to be a valid and reliable instrument for assessing positive MHL in the Norwegian adolescent population [35]. The results should be interpreted with caution considering some limitations. The subsample of adolescents who were matched in the cohort was small. This smaller size might be due to the teachers handing out the questionnaire to different adolescents at baseline T1 and T2. Furthermore, the six-letter code questions might not have performed optimally, resulting in difficulties matching adolescents from baseline T1 to T2. The lack of the possibility to randomize the adolescents participating in MEST is a limitation of the study. MEST participation was assessed by self-report; thus, assignment to the group that received MEST or the group that did not receive MEST in the average treatment effect models was based on recall of intervention (MEST), which may be subject to recall bias. Further, all variables in the current study were self-reported; family finances were represented by students’ perception of family finances, and parents’ level of education was reported by students. School nurses did not report procedural fidelity to MEST. The flexible nature of MEST as a working strategy rather than a procedure can be considered a strength of MEST, but it also makes it more challenging to evaluate and, as such, is a limitation of the current study. Furthermore, schools are complex settings; thus, other mental health-promoting activities occurred as part of the daily operations of the schools over the school year, making it difficult to attribute changes to MEST. However, these activities and programs were offered to both students who participated in MEST and those who did not participate. Another limitation is the lack of measurement of the dose of MEST (i.e., how many seminars the participating adolescents attended). Thus, there may be differences in outcome between students who completed several sessions of MEST compared to students participating in only one session, that this study was not able to detect. Furthermore, HL was only measured at T2; thus, baseline T1 HL was not controlled for, although baseline MHL was measured and controlled for. Furthermore, there may be confounders that were not accounted for with respect to mental wellbeing and positive MHL. However, the included covariates were based on factors that are known to affect the outcome variables. The current study may serve as a foundation for further evaluations of MEST, although no causal relationship can be established at this point. As we confront challenges with mental health problems in the adolescent population, utilizing well-performing and evaluated interventions and work strategies is vital for promoting mental health in this population. This study indicates that further investment in the evaluation of the newly developed work strategy MEST is warranted. Although positive results were found in this initial study of MEST, this working strategy must be further evaluated to establish its effect and feasibility. Important next steps include conducting a thorough evaluation of MEST and its implementation. Furthermore, additional investigations of gender differences in MEST attendance, evaluations of the effect of MEST separately by gender, and potentially developing MEST to be equally appealing to both genders are needed.

## Conclusions

Overall, the results of the descriptive statistics and ATE models of MEST expand our knowledge of MEST and how it affects positive MHL and mental wellbeing among adolescents. Modeling the ATE of MEST showed that MEST participants had a higher level of positive MHL compared to the non-MEST participants and that girls who participated in MEST had higher levels of mental wellbeing compared to non-participating girls. No differences in the ATE of MEST on boys’ mental wellbeing were identified. Although this study cannot be used as evidence to recommend MEST, the current results provide a foundation for recommending further investments in more rigorous and resource-intensive evaluations of MEST as a work strategy for school health services addressing adolescent mental health. The results from the study may contribute in the comprehensive picture of mental health promotion work and the evidence base for school health services. Further studies should include evaluating the effect of MEST on positive MHL and mental wellbeing in a randomized controlled trial as well as investigating gender differences and the implementation and feasibility of MEST.

## Abbreviations

AIPW: Augmented Inverse Probability Weighting
ATE: Average treatment effect
MHL: Mental health literacy
MHPK: Mental health-promoting knowledge
POM: Potential outcome mean
SWEMWBS: Short Warwick-Edinburgh Mental Wellbeing Scale
